# Clinically Probable Late-Onset Familial Mediterranean Fever Presenting as Recurrent Pericarditis and Suggestive of Evolving Constrictive Physiology

**DOI:** 10.7759/cureus.110198

**Published:** 2026-06-03

**Authors:** Dhruva Govil, Ahmad Ather, Keyur Patel

**Affiliations:** 1 Internal Medicine, Henry Ford Providence Hospital, Southfield, USA; 2 Internal Medicine, Henry Ford Health System, Southfield, USA

**Keywords:** autoinflammatory disorder, colchicine, familial mediterranean fever, pleural effusion, recurrent pericarditis

## Abstract

Familial Mediterranean fever (FMF) is a rare genetic autoinflammatory disorder that usually appears in childhood. It is marked by recurrent episodes of fever and inflammation of the serous membranes. Late-onset cases are rare and often misdiagnosed due to symptoms similar to those of other infections or inflammatory diseases. We report the case of a 65-year-old man who presented with recurring fever, pleural effusions, and pericarditis over two months, suggestive of evolving constrictive physiology. Despite being hospitalized several times, tests for infections and cancers ruled out other causes. This included thoracenteses, a pericardial window, a pleural biopsy, and bone marrow analysis. Pathology showed fibrinous pericarditis and chronic pleuritis with fibrosis, without signs of cancer. The patient was started on colchicine, which led to a quick improvement in his condition. Considering the symptom presentation and response to colchicine, we diagnosed clinically probable FMF based on established criteria, noting that late-onset presentations over the age of 60 represent fewer than 5% of all FMF cases. This case emphasizes the need to suspect FMF in patients with unexplained recurrent serositis, even in older individuals. Although heart involvement in FMF is uncommon, it can imitate serious conditions and delay proper treatment. Colchicine is the main treatment, and regular follow-ups are important to check for response and side effects. A team approach is vital, especially in unusual cases, to lower the risk of complications such as amyloidosis and heart problems.

## Introduction

Familial Mediterranean fever (FMF) is a rare autoinflammatory disorder primarily affecting individuals of Mediterranean descent and is caused by mutations in the *MEFV* gene. It is characterized by recurrent, self-limited episodes of fever and serosal inflammation, most commonly involving the peritoneum, pleura, and synovium [1-3]. Cardiac involvement is uncommon and often under-recognized, particularly in older adults. Although FMF is typically diagnosed in childhood or early adulthood, late-onset presentations can pose a significant diagnostic challenge, especially when clinical features overlap with infectious, malignant, or other inflammatory etiologies. Pericarditis is a rare but well-described manifestation of FMF and may be recurrent or refractory, leading to invasive diagnostic procedures if the underlying autoinflammatory etiology is not recognized [4,5].

This case report illustrates the diagnostic journey of a 65-year-old male with recurrent fever, pleural effusions, and pericarditis suggestive of evolving constrictive physiology, who was ultimately diagnosed with clinically probable FMF after an extensive and multidisciplinary evaluation. To guide the clinician before formal criteria application, the diagnostic instrument used later in this evaluation is the classic Tel Hashomer criteria set. It is critical to note that late-onset FMF remains exceptionally rare, accounting for an estimated 2%-5% of cases globally, and its diagnosis is frequently confounded by the fact that recurrent idiopathic pericarditis itself shares an excellent response to empiric colchicine therapy.

## Case presentation

A 65-year-old male with a past medical history of hypertension and chronic venous stasis presented with recurrent episodes of fever, pleuritic chest discomfort, and progressive dyspnea over a two-month period. The patient experienced a frequency of approximately three to four distinct episodes over this two-month span, with each symptomatic attack lasting roughly 48 to 72 hours before partial spontaneous defervescence. Regarding epidemiological context, the patient reported no known family history of recurrent fevers, unexplained serositis, or documented FMF. He required multiple hospital admissions for fever of unknown origin and recurrent bilateral pleural effusions, initially treated as presumed infectious pneumonia. Despite broad-spectrum antibiotics and repeated thoracenteses, symptoms recurred.

Initial laboratory evaluation revealed elevated inflammatory markers, including C-reactive protein and erythrocyte sedimentation rate, with otherwise unremarkable complete blood count and metabolic panel. Comprehensive infectious, autoimmune, and malignant evaluations were unrevealing, including blood cultures, tuberculosis testing, autoimmune serologies, and cytology from pleural fluid (Table 1). Serum amyloid A (SAA), ferritin, and procalcitonin levels were not obtained during the acute presentation window.

**Table 1:** Initial laboratory results

Lab	Value	Reference range
C-reactive protein (CRP)	199.6 mg/L	1-10 mg/L
Erythrocyte sedimentation rate (ESR)	94 mm/h	0-20 mm/h
Lactic acid	1.20 mmol/L	0.50-2.00 mmol/L
Quantiferon-TB Gold Plus	Negative	Negative
Antinuclear antibody (ANA)	Negative	Negative
IgG total	889 mg/dL	610-1616 mg/dL
Blastomycosis antibody	Negative	Negative
*Aspergillus fumigatus* IgG	Negative	Negative
Aureobasidium pullulans	Negative	Negative
HIV Ab 1 and 2	Non-reactive	Non-reactive
HIV-1 p24 antigen	Non-reactive	Non-reactive
White blood cells (WBCs)	6.59 K/mcL	4-11 K/mcL

Transthoracic echocardiography demonstrated a small-to-moderate circumferential pericardial effusion with septal bounce and respiratory variation of tricuspid inflow, raising concern for evolving constrictive physiology (Figure 1, Appendix A). Pulsed-wave Doppler of the tricuspid valve inflow demonstrated a 33% increase in early diastolic (E-wave) velocity during inspiration, without evidence of right atrial or right ventricular diastolic collapse. Computed tomography of the chest also revealed a small pericardial effusion with pericardial wall thickening (Figure 2). These findings, in conjunction with pleuritic chest pain and elevated inflammatory markers, supported the diagnosis of recurrent pericarditis.

**Figure 1:**
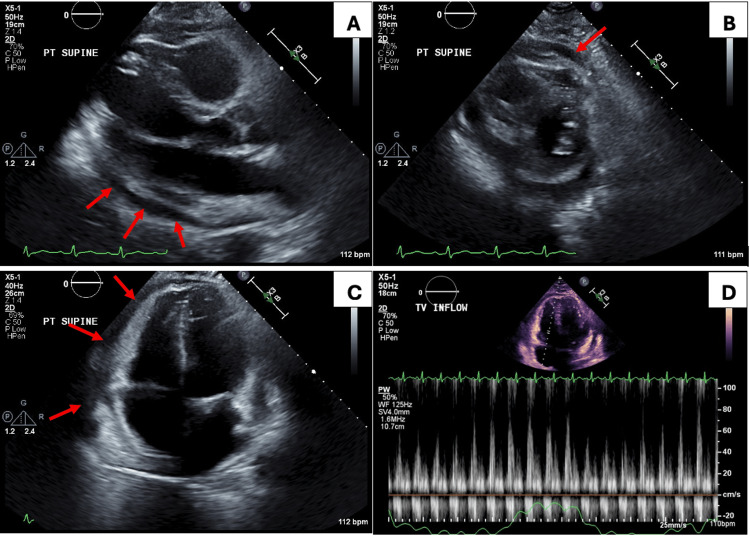
Transthoracic echocardiography Demonstration of circumferential pericardial effusion with respirophasic inflow variation. (A-C) Two-dimensional imaging reveals a small-to-moderate circumferential pericardial effusion (red arrows) in the (A) parasternal long-axis, (B) parasternal short-axis, and (C) apical four-chamber views. There is no evidence of right atrial or right ventricular diastolic collapse in these views. (D) Pulsed-wave Doppler of the tricuspid valve inflow demonstrates a 33% increase in early diastolic (E-wave) velocity during inspiration.

**Figure 2:**
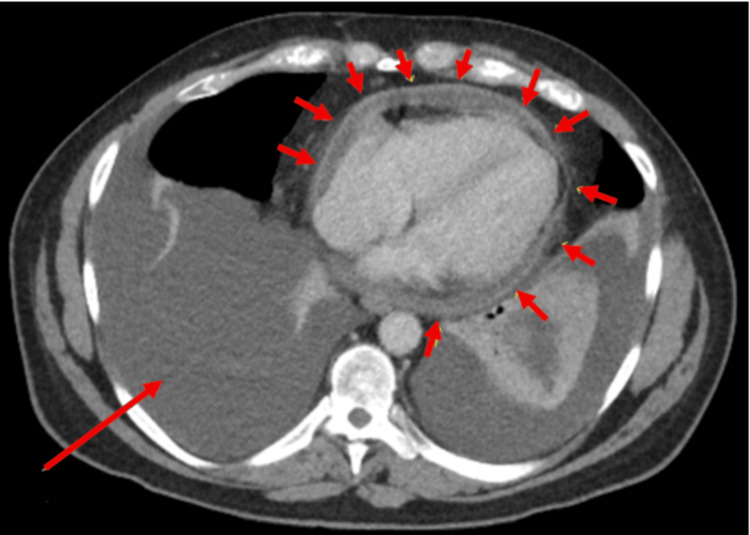
Computed tomography of the chest Moderate pericardial effusion with pericardial thickening and enhancement (short arrow) suggesting pericarditis. Bilateral pleural effusions with compression atelectasis of the lower lung lobes bilaterally, larger on the right (long arrow).

Given persistent fevers, recurrent serositis, and imaging evidence suggesting evolving constrictive pericarditis, the patient underwent a surgical pericardial window, along with anterior mediastinal lymph node dissection and right pleural biopsy. Histopathology revealed fibrinous pericarditis and chronic fibrosing pleuritis without evidence of malignancy or infection.

Despite an extensive evaluation, including bone marrow biopsy and flow cytometry, no alternative etiology was identified. Formal *MEFV* genetic testing was deferred at the time of evaluation due to institutional resource constraints and a lack of immediate local availability, rendering this a clinical-by-necessity diagnostic scenario. The constellation of recurrent fever, pleuritis, pericarditis, and dramatic clinical response to colchicine prompted consideration of an autoinflammatory disorder. The patient met one major criterion (recurrent serositis presenting as unilateral pleuritis and pericarditis) and two minor criteria (recurrent fevers and a favorable response to colchicine) consistent with the Tel Hashomer criteria (Table 2). According to the validated decision rule, the presence of at least one major criterion or two minor criteria within this system is sufficient to establish a clinical diagnosis of FMF.

**Table 2:** Tel Hashomer criteria for familial Mediterranean fever diagnosis criteria Criteria adapted from the Sohar/Pras framework [6]

Major criteria
Typical attacks
1. Peritonitis (generalized)
2. Pleuritis (unilateral), pericarditis, serositis
3. Monoarthritis (hip, knee, ankle)
Minor criteria
1-3. Incomplete attacks involving 1 or more of the following sites:
1. Abdomen
2. Chest
3. Joint
4. Recurrent fevers
5. Favorable response to colchicine

The patient was initiated on colchicine 0.6 mg twice daily, resulting in rapid resolution of fever (with complete defervescence observed within 24 hours), improvement in dyspnea, and subsequent normalization of inflammatory markers. At the point of colchicine initiation, the patient's baseline renal and hepatic function was carefully documented, demonstrating a serum creatinine of 0.92 mg/dL (estimated glomerular filtration rate >60 mL/min/1.73 m^2^) and normal transaminases, and a thorough CYP3A4 and P-glycoprotein (P-gp) drug interaction review was performed to confirm the safety of co-prescribing with his current antihypertensive regimen.

He was discharged with close rheumatology and cardiology follow-up. Per the European League Against Rheumatism (EULAR) 2016 recommendations for the management of FMF [7], he was scheduled for follow-up every six months to monitor treatment response, clinical toxicity, and inflammatory markers; routine SAA monitoring will be executed at each six-month interval as part of this EULAR-aligned surveillance plan to proactively mitigate long-term amyloidosis risks.

## Discussion

FMF is an autoinflammatory disease caused by mutations in the *MEFV* gene, leading to dysregulation of the inflammatory response. While serositis is common in FMF, cardiac involvement, particularly pericarditis, is less frequently observed but can present significant clinical challenges. Recurrent serositis remains the hallmark of the disease and was first characterized in large Mediterranean cohorts, where peritonitis and pleuritis predominated. Although cardiac involvement is less common, pericarditis has been reported and may occur as part of the inflammatory spectrum of FMF [1].

Research indicates that cardiovascular autonomic dysfunction can occur in FMF patients, particularly those who are colchicine-responsive. Rozenbaum et al. noted that patients with FMF may experience such dysfunction, which can further complicate the disease course [1]. Additionally, studies by Canpolat et al. have demonstrated the importance of evaluating cardiac autonomic indices in FMF patients on colchicine treatment, highlighting the need for careful cardiac monitoring in these patients [3]. Furthermore, Nussinovitch et al. explored the variability of the QT interval in FMF patients, noting differences between those who were colchicine-responsive and those who were colchicine-resistant. This variability underscores the complex cardiac implications of FMF and the necessity of tailored therapeutic approaches [4,8].

Another study by Gang et al. emphasized the role of cytokine activation in FMF, which can contribute to both the systemic and cardiac manifestations of the disease [5]. The continuity of cytokine activation in FMF patients was further explored by Bagci et al., who highlighted a persistent inflammatory state, even during colchicine treatment. This ongoing inflammation may play a role in the development of recurrent pericarditis and other serosal involvements [9]. Lastly, Erken et al. discussed the potential benefits of early suppression of FMF attacks using medium-dose methylprednisolone, suggesting that early intervention may help in managing severe cases and preventing long-term complications [10].

To contextualize these manifestations within the broader medical literature, Aydin et al. examined a large single-center late-onset FMF series encompassing 41 patients with disease onset after age 40 (representing 2.02% of their total cohort), demonstrating that late-onset presentations typically feature a milder phenotype, lower required maintenance colchicine doses, and a significantly lower prevalence of homozygous exon 10 mutations [11]. Furthermore, the comprehensive cardiac review by Alsarah et al. reports that while pericarditis is a recognized manifestation of FMF, it remains an uncommon primary presentation, usually presenting as a transient, benign effusive process rather than a chronic constrictive or restrictive physiology [12]. The specific incremental teaching point of this case lies in its presentation at age 65 -- exceeding the standard baseline boundaries of the Aydin cohort -- featuring an isolated pericardial effusion with constrictive features, which prompted an extensive invasive surgical workup before the initiation of a successful trial of colchicine.

This body of literature reinforces the complexity of FMF, particularly in patients with atypical presentations such as recurrent pericarditis. A multidisciplinary approach, involving rheumatology, cardiology, and genetics, is crucial for managing such cases. Early diagnosis and treatment with colchicine are essential to prevent severe complications, including amyloidosis and cardiac dysfunction, as highlighted by these studies [11,12].

This case report has several notable limitations. First, the diagnosis of FMF is genotype-unconfirmed, as formal molecular testing of the *MEFV* gene was unavailable at our institution, creating a reliance on clinical diagnostic rules. Second, clinical and biochemical responsiveness to colchicine is not fully specific to FMF; as demonstrated by the extensive evidence base from the ICAP and CORP clinical trials, colchicine is a primary, highly effective therapeutic pillar for idiopathic recurrent pericarditis, which represents a major alternative diagnosis in this patient's differential diagnosis. Third, the patient's constrictive physiology was strongly suggested by non-invasive imaging findings (echocardiographic septal bounce and respirophasic tricuspid variation) rather than definitively confirmed via simultaneous right and left ventricular cardiac catheterization. Finally, this evaluation relies entirely on clinical criteria sets, which carry well-documented diagnostic limitations and lower baseline predictive values when applied to highly atypical, late-onset presentations in older adults.

## Conclusions

This case highlights clinically probable late-onset FMF presenting with recurrent pericarditis suggestive of evolving constrictive physiology, emphasizing the importance of maintaining a broad differential diagnosis in patients with unexplained recurrent serositis. Prompt clinical recognition and early initiation of colchicine can lead to rapid clinical improvement and may prevent future unnecessary invasive procedures and long-term complications in similar presentations.

## Figures and Tables

**Video 1:** Transthoracic echocardiography

## Appendices

Appendix A 
